# Supplementary material for: Male pheromones modulate synaptic transmission at the C. elegans neuromuscular junction in a sexually dimorphic manner
Source: eLife. 2021 Mar 31;10:e67170. doi: 10.7554/eLife.67170 (PMC8051947; doi:10.7554/eLife.67170)
Supplement: Supplementary file 1. [file elife-67170-supp1.docx]

| **Supplementary File 1: *C. elegans* strains used in this study** | | |
| --- | --- | --- |
| **Strain** | **Genotype** | **References** |
| N2(Bristol) |  | CGC |
| AB3 |  | CGC |
| TR389 |  | CGC |
| CB4856 |  | CGC |
| RB1562 | *him-5(ok1896) V* | CGC |
| PR691 | *tax-2(p691) I* | CGC |
| CX3222 | *odr-3(n1605) V* | CGC |
| VC1785 | *acox-1.1(ok2257) I* | CGC |
| VS18 | *maoc-1(hj13) II* | CGC |
| TM2581 | *dhs-28(tm2581) X* | NBRP |
| RB859 | *daf-22(ok693) II* | CGC |
| TXJ0938 | *daf-22(ok693); him-5(ok1896)* | In this study |
| VC854 | *unc-2(gk366) X* | CGC |
| TXJ0819 | TR389;*him-5 (xj001)V* | In this study |
| TXJ0583 | *tax-2(691) I;* xjEx0036[Pstr-1::tax-2 + Plin-44::GFP] | In this study |
| TXJ0611 | *tax-2(691) I;* xjEx0038[Pdaf-28::tax-2 + Plin-44::GFP] | In this study |
| TXJ0616 | *odr-3(n1605) V;* xjEx0052[Pstr-1::odr-3 + Plin-44::GFP] | In this study |
| TXJ0595 | xjEx0005[Pflp-21::tomm-20N-miniSOG + Pflp-21::mCherry + Pmyo-2::eGFP] | In this study |
| TXJ0620 | xjEx0011[Pceh-36::tomm-20N-miniSOG + Pmyo-3::mCherry] | In this study |
| TXJ0581 | xjEx0033[Pdaf-28::tomm-20N-miniSOG + Pdaf-28::mCherry + Pmyo-2::eGFP] | In this study |
| TXJ0621 | xjEx0012[Pstr-1::tomm-20N-miniSOG + Pstr-1::mCherry + Pmyo-2::eGFP] | In this study |
| TXJ0577 | xjEx0016[Psra-7::tomm-20N-miniSOG + Psra-7::mCherry + Pmyo-2::eGFP] | In this study |
| TXJ0622 | xjEx0004[Pgpa-4::tomm-20N-miniSOG + Pgpa-4::mCherry + Pmyo-2::eGFP] | In this study |
| TXJ0588 | xjEx0021[Psrb-6::tomm-20N-miniSOG + Psrb-6::mCherry + Plin-44::GFP] | In this study |
| TXJ0591 | xjEx0023[Pgcy-15::tomm-20N-miniSOG + Pgcy-15::mCherry + Plin-44::GFP] | In this study |
| TXJ0629 | xjEx0056[Podr-10::tomm-20N-miniSOG + Podr-10::mCherry + Plin-44::GFP] | In this study |
| TXJ0566 | xjSi0004[Pdaf-28::oCHIEF::mCherry] | In this study |
| TXJ0567 | xjSi0005[Pstr-1::oCHIEF::mCherry] | In this study |
| TXJ0761 | xjSi0007[Pstr-2::oCHIEF::mCherry] | In this study |
| TXJ0775 | xjSi0008[Podr-10::oCHIEF::mCherry] | In this study |
| TXJ1052 | xjEx0058[Pstr-1::GCaMP6f + Pstr-1::mCherry + Plin-44::GFP] | In this study |
| TXJ0630 | xjIx0004[Pacr-5:: ChrimsonN::mCherry]； ljIs131[Pmyo-3::GCaMP3] | In this study |
| ZM7982 | ljIs131[Pmyo-3::GCaMP3::tagRFP] | Shangbang Gao |
| KP5931 | nuIs283[Pmyo-3::unc-49::GFP; Punc-25:: RFP::rab-3] | Joshua Kaplan |
| KP6221 | nuIs431[Punc-129::GFP::rab-3] | Joshua Kaplan |
| TXJ0502 | xjSI0002[Pmyo-3::acr-16::RFP] | In this study |
| kr208 | kr208[Punc-29::unc-29::tagRFP] | Jean-Louis Bassere |
| EN2630 LGII | *unc-49(e407);* krSi2[Punc-49::unc-49B::RFP] | Jean-Louis Bessereau |
| KP9809 | nu586[unc-2::GFP11x7]; nuSi250[Punc-129::splitGFP1-10::sl2::unc57-mCherry::sl2::mTagBFP2] | Joshua Kaplan |
| KP9639 | nu586[unc-2::GFP11x7]; nuSi251[Punc-47::splitGFP1-10::sl2::unc57-mCherry::sl2::mTagBFP2] | Joshua Kaplan |
