# Supplementary material for: Male pheromones modulate synaptic transmission at the C. elegans neuromuscular junction in a sexually dimorphic manner
Source: eLife. 2021 Mar 31;10:e67170. doi: 10.7554/eLife.67170 (PMC8051947; doi:10.7554/eLife.67170)
Supplement: Supplementary file 2. [file elife-67170-supp2.docx]

| **Supplementary File 2: Sequence information** | |
| --- | --- |
| **Cloning Primers** | |
| P*ceh-36* F | tagaactcccgcagaatgccaac |
| P*ceh-36* R | tgtgcatgcgggggcaggcgaagtgct |
| P*odr-10* F | tgactcataaatcaataccagtctg |
| P*odr-10* R | ggagctgtaaggtatcttaa |
| P*str-1* F | agaaccactacacttgaacgatacgaa |
| P*str-1* R | tagtcaaatgatatgaagtttgtgttaaga |
| P*srb-6* F | tctacttttaaatattatatctttc |
| P*srb-6* R | ttttatttcttctgtagaaatttcaag |
| P*gpa-4* F | ggatccattctcaaaatcgcagaagtc |
| P*gpa-4* R | tgttgaaaagtgttcacaaaatg |
| P*gcy-15* F | ccatgacgacgcttgatatgttc |
| P*gcy-15* R | agctgatgggatgtaggcagcac |
| P*sra-7* F | agacgacatgatctagatgactctag |
| P*sra-7* R | ggcttctaatatttcgagaaactgc |
| P*flp-21* F | tgaggtcacgcaacttgatga |
| P*flp-21* R | gaaaatgactttttggattttgga |
| P*acr-5* F | atttgttgaaaaaacgtacggtcttc |
| P*acr-5* R | gctgaaaattgtttttaaagcattg |
| P*myo-3* F | cccgacaaaacatgagtatttc |
| P*myo-3* R | ccctctagatggatctagtg |
| P*unc-25* F | Agagaaaagcgcttcataagacg |
| P*unc-25* R | ttttggcggtgaactgagcttttc |
| P*unc-129* F | gaaacatgatatcgacggacata |
| P*unc-129* R | cttgcttgctcttccaattttcctg |
| P*str-2* F | atataaatcaatgggatcaacgcc |
| P*str-2* R | ttttatggatcacgagtattcg |
| TAX-2(F36F2.5.1) F | atgtatcaagttccaaaacgagca |
| TAX-2(F36F2.5.1) R | ttaatcggcatgtagtttctgtgttcc |
| ODR-3(C34D1.3.1) F | atgggctcatgccagagc |
| ODR-3(C34D1.3.1) R | ttacatcattcctgctttttgtaaattcttctg |
| **Genotyping primers** | |
| *unc-2*-GFP_11_ F | ggattgttaacggaggagtagg |
| *unc-2*-GFP_11_ Rin | ctcgtgaagaaccatgtgatc |
| *unc-2*-GFP_11_ Rout | ctaaacaattgcccatcgagga |
| *him-5(xj001)* F | actacttcctaaatccaatccagg |
| *him-5(xj001)* R | agcttcattcactacttcgtc |
| **CRISPR information** | |
| TR389;*him-5(xj001)* mutant was deleted 5 bases on the second exon of D1086.4a.1 | aacagttggtcgc<atcgc>cggtcgttcaca |
